# The use of virtual reality in forensic‐correctional psychiatric settings: A systematic review

**DOI:** 10.1111/pcn.70068

**Published:** 2026-04-29

**Authors:** Michael Y. Wang, Eric Yu, Rida Tauqir, Maya Mckeown, Shahnawaz Towheed, Mark M. Kaggwa, John M. Bradford, Gary A. Chaimowitz, Andrew T. Olagunju

**Affiliations:** ^1^ Michael G. DeGroote School of Medicine McMaster University Hamilton Ontario Canada; ^2^ Department of Psychiatry and Behavioural Neurosciences, Faculty of Health Sciences McMaster University Hamilton Ontario Canada; ^3^ Forensic Psychiatry Program, St. Joseph's Healthcare Hamilton Hamilton Ontario Canada; ^4^ Department of Psychiatry University of Toronto Toronto Ontario Canada; ^5^ Discipline of Psychiatry Adelaide University Adelaide South Australia Australia; ^6^ Department of Psychiatry and Behavioral Sciences University of Oklahoma Oklahoma City Oklahoma State USA; ^7^ Federal Neuropsychiatric Hospital Calabar Cross River State Nigeria

**Keywords:** corrections, forensics, mental health, technology, virtual reality

## Abstract

Virtual reality (VR) is increasingly being used as an innovative technology for assessment, treatment, and training within psychiatric settings. However, little work has been done to synthesize existing literature on the use and benefits of VR in forensic‐correctional settings. This review was conducted in accordance with the Preferred Reporting Items for Systematic Reviews and Meta‐Analyses guideline. Protocol is available at Open Science Framework (https://osf.io/3m6p2). A total of 1317 articles were screened, and 32 reports were included in the final review. VR interventions were identified as effective in forensic‐correctional settings for assessment of patients with pedophilic disorders, reducing aggression and stress, increasing empathy in intimate partner violence (IPV) offenders, enhancing community reintegration through education and vocational readiness, and improving patient engagement. Furthermore, VR allows innovative incorporation of biophysiological measures to support objective and quantifiable measures of risk within forensic‐correctional populations. Barriers to VR use and implementation included cybersickness in users, the knowledge and technical burden to utilize interventions, and the demand for resources. The quality of the included studies was assessed as predominantly moderate. Overall, the findings in this review highlight the promising benefits of VR as an innovative tool for assessments and care in forensic‐correctional settings. There is need for stakeholders' support, standardization of VR uses, and promotion of evidence‐based guidelines for successful implementation and effective integration into care. As the integration of technologies into healthcare continues to advance, VR has the capacity to reshape mental health care within forensic‐correctional settings and future research is needed to guide effective use.

Globally, virtual reality (VR) is increasingly recognized as a powerful technological tool with innovative applications in multiple fields, including gaming, education, healthcare, manufacturing and military among others.^1^, ^2^, ^3^, ^4^, ^5^ VR uses computer‐generated images and sounds to create an interactive environment through which scenarios are simulated to engage users.^6^, ^7^, ^8^, ^9^ While VR can be either immersive or non‐immersive, the immersive VR solutions allow users to engage with a virtual environment wearing specialized equipment such as a head‐mounted display (HMD) or a Cave Automatic Virtual Environment system (CAVE).^9^ In non‐immersive VR, users engage with virtual environments and scenarios through more traditional interfaces such as a computer or video game console with a display.^9^ Both immersive and non‐immersive VR have been used effectively across a variety of settings, albeit the level of immersion and realism is positively correlated with the users' benefits.^6^, ^7^, ^8^, ^9^

Multiple lines of evidence show that VR is gaining traction as an innovative interventional tool in healthcare settings.^4^, ^5^, ^10^ For instance, VR‐based interventions are utilized to manage chronic and procedural pain,^11^ assist in balance and gait rehabilitation post‐stroke,^12^ improve symptom burden in patients with terminal cancer,^13^ and facilitate medical education and clinical decision making.^4^, ^5^, ^14^ VR has also made significant strides in psychiatry with current literature indicating that VR is effective in the treatment of anxiety disorders, depression, stress‐related disorders, phobias, obesity and eating disorders, substance use disorders, conduct disorder, and oppositional defiant disorder.^15^, ^16^, ^17^, ^18^ The efficacy of VR for delivering psychosocial treatments for many of the aforementioned psychiatric illnesses is partly linked to its ability to introduce realistic stimuli in a controlled environment while simultaneously monitoring patient responses, thereby allowing patients to practice therapeutic techniques in an ethical and realistic setting.^15^, ^16^, ^18^ With substance use disorder specifically, one noteworthy benefit of VR is the ability to incorporate social interactions as stimuli, since social interactions themselves can be craving agents and an incentive to consume such substances.^16^ VR also provides patients with diverse psychiatric conditions the opportunity to gain exposure to higher levels of stimuli than possible in real‐world therapies, yielding increased perceptions of self‐efficacy and consequently more positive patient outcomes.^18^ In the same vein, VR has the unique ability to seamlessly integrate various psychotherapeutic techniques, such as cognitive behavioral therapy, exposure therapy, and classical conditioning, for the optimization and customization of therapy regimens.^19^

Despite the growing interest in VR as a therapeutic tool within the field of psychiatry, its use within forensic and correctional psychiatry, a specialty of psychiatry designed to assess and care for individuals in the criminal justice system with mental disorders,^20^, ^21^, ^22^, ^23^, ^24^ is still at an early stage. However, it stands to reason that many established applications of VR within general psychiatry would likely be relevant in forensic and correctional psychiatric settings. This is especially true given that forensic‐correctional psychiatric settings are known to care for individuals with a high burden of severe and persistent symptoms of mental illnesses, personality disorders, and substance use requiring innovative psychotherapeutic techniques with demonstrable effectiveness.^16^, ^18^, ^25^, ^26^, ^27^ Moreover, the population in forensic‐correctional psychiatric settings often include individuals with unique attributes (e.g. sexual offense, risk of violence, psychopathy, recidivistic risk) and a myriad of challenges that are better explored and treated in a simulated environment due to their special risk issues, safety and security concerns, and ethical dilemmas.^20^, ^22^, ^27^, ^28^, ^29^

At present, little work has been done to consolidate the existing evidence for the applications and benefits of VR within forensic‐correctional psychiatry. Notably, previous reviews have a relatively limited scope of the current VR interventions that have been directly utilized within forensic‐correctional psychiatric settings, thereby having scanty information on recent developments on the use of VR within the field.^24^ Our review thus addresses the pivotal need to synthesize the existing literature, reflecting recent trends in the field. Through this study, we aimed to explore the benefits of using virtual reality in patients' assessment and care on specific outcomes, such as improving mental health symptoms, risk mitigation and community reintegration. Furthermore, we seek to identify the current challenges of implementing VR and make appropriate recommendations to promote evidence‐based guidelines and interventions in the use of VR in forensic‐correctional populations.

## Methods

### Search strategies and article screening

This review was conducted in accordance with the Preferred Reporting Items for Systematic Reviews and Meta‐Analyses guidelines (PRISMA).^30^ The review protocol was published in Open Science Framework.^1^ A systematic search was conducted to identify relevant articles in major databases (MEDLINE/PubMed, PsycINFO, Web of Science, and Embase). The search for potential articles was conducted using Medical Subject Headings and keywords, which were placed into the following categories: (i) Virtual Reality, (ii) Corrections, (iii) Forensic. The database search was supplemented with a snowball search for relevant studies. Study authors were contacted where necessary to request their work. All identified articles were exported into Covidence and duplicates were removed. Screening of title, abstract, and full‐text review of all identified articles was then conducted by at least two independent investigators and conflicts were resolved by discussion between the investigators or with consultation with the senior author on the paper where necessary. Details on the search strategy are included in the supplementary document S1a and S1b.

### Selection of eligible studies

We considered studies that examined the use of VR‐based interventions in forensic‐correctional settings. Studies were included based on the criteria that: (i) they were peer‐reviewed original studies, (ii) consisted or focused on at least one VR‐based intervention (including both immersive and non‐immersive). Articles were excluded if they: (i) did not include relevant interventions and population groups, (ii) consisted of case report, conference abstract, editorials, book chapters, protocols, or unpublished report, (iii) were incomplete or did not have available full text, or (iv) were not available in the English language.

### Data extraction and synthesis

Study information was collected with a data extraction table including items such as the name of the author, year of publication, characteristic of the study population, setting of the study, type of study/design, characteristics of the VR intervention, characteristics of non‐VR interventions, behavioral conditions/diagnosis (e.g. psychosis, substance abuse and other mental health conditions), mental health problems/outcomes, educational outcomes, quality of life/wellbeing outcomes, and other findings. The data extracted on main findings included the type of VR intervention used, description of the study population, measures used for assessments, and information on outcomes and specific study‐defined interventions. The data extraction process was completed by at least two independent investigators and conflicts were resolved by discussion between the investigators and when necessary with consultation with the senior author. The extracted information addressed most of the specific aims of this systematic review and manual thematic analysis was used to synthesize the results.

### Critical assessment of quality and bias

A quality assessment was conducted for both qualitative and quantitative studies. For qualitative studies, the Critical Appraisal Skills Programme (CASP) framework was utilized.^31^ For quantitative studies, the National Institutes of Health (NIH) Study Quality Assessment Tools were utilized to evaluate eligible studies on a range of 12–14 items depending on their designs.^32^, ^33^ Studies with mixed methods were evaluated using both tools. The quality of each study was then classified into three categories: low (below 40%), moderate (40–75%), and high (above 75%), based on the percentage of the criteria met. Scores were calculated by dividing the number of positive responses by the total criteria used. At least two independent authors conducted the evaluations, and the average of their scores was used for the final assessment.

## Results

### Screening and selection of eligible articles

**Fig. 1:**
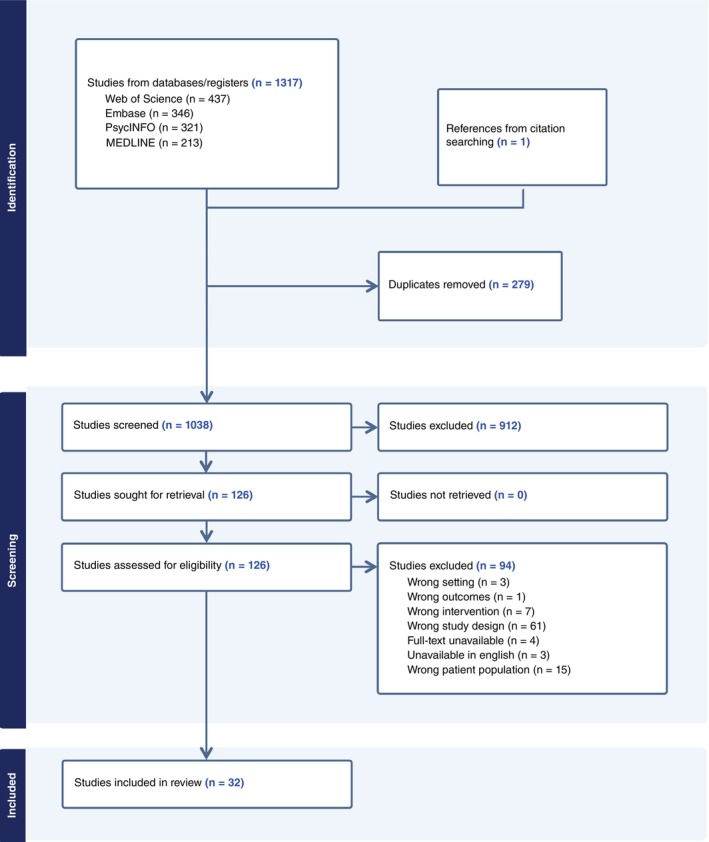
PRISMA flow diagram.

A total of 1317 articles were originally identified, with 1038 articles progressing to title and abstract screening following removal of duplicates. This screening yielded 126 studies for full‐text review and 32 reports were eligible for inclusion in the final review.^34^, ^35^, ^36^, ^37^, ^38^, ^39^, ^40^, ^41^, ^42^, ^43^, ^44^, ^45^, ^46^, ^47^, ^48^, ^49^, ^50^, ^51^, ^52^, ^53^, ^54^, ^55^, ^56^, ^57^, ^58^, ^59^, ^60^, ^61^, ^62^, ^63^, ^64^, ^65^ Figure 1 shows the PRISMA flow diagram, which shows the article selection process in detail.

### Characteristics of the included studies

The included studies (n = 32) were conducted in multiple countries, including the Netherlands (n = 10), the USA (n = 4), Spain (n = 3), Sweden (n = 6), Canada (n = 2), New Zealand (n = 2), the United Kingdom (n = 2), Australia (n = 1), Germany (n = 1), and Saudi Arabia (n = 1).

Included studies were of quantitative (n = 20), qualitative (n = 6), and mixed methods (n = 6) in nature, with various types of quantitative study (trials, cohort, case study, etc.) included in the review.

Sample sizes in the included studies ranged widely largest sample size was 197, with a total of 1197 individuals assessed across all 32 included studies. A majority of the studies were published in the year 2020 or later (n = 23), seven studies were published between 2010 and 2019 (n = 7) and only 2 studies were published prior to 2010. The study populations included offending individuals with mental illnesses held in forensic hospital wards, long‐term intensive psychiatric hospitals, and prisons (n = 826, 69.01%). Some studies reported the perspectives of healthcare workers, including psychiatrists, psychologists, coordinating therapists, team managers, forensic nurses, and socio‐therapists (n = 69, 5.76%). Some studies also assessed healthy controls (n = 286, 23.89%). Detailed characteristics of all 32 included studies are presented in Table 1.

**Table 1:** Study characteristics and findings

Author, Year & Country	Study design	Characteristics of the study population	VR technology and study intervention	Main findings
Fromberger *et al*.,^40^ 2018, Germany	CC*	Six SOCs (age = 47.67, SD = 13.47) in a forensic hospital ward. All SOCs perpetrated at least one documented sexual assault against children. Recidivistic risk was moderate to high, and no unsupervised hospital privileges outside the ward. Seven NOCs with no history of neurological or psychological illness. (age = 26.00, SD = 4.36) were recruited *via* posters or social media.	The VR system used a head‐mounted display and a motion capturing system Participants rated the attractiveness of virtual characters followed by three virtual risk scenarios where participants interacted with a child character. The experiment allowed therapists to monitor behavior of SOCs, without endangering the community, to assess their decision making	Primary Outcome: In 89% of SOCs, their response behavior did not correspond to their own beliefs of correct behavior. In 62% of SOCs, their behavior did not correspond to the coping skills they stated that the therapists focused on in therapy. In 50% of SOCs, response behavior corresponded to coping skills therapists reported they focused on in therapy. Therapists were correct in predicting the behavior of SOCs in 75% of virtual risk scenarios. Secondary Outcome: Indicators of Immersion including subjective feelings of presence (sense of physically being in the virtual environment) was reported to be high and co‐presence (sense of virtual character being in your environment) was reported to be medium in all experimental conditions. Subjective realism of virtual scenes was rated highly. Simulator sickness symptoms were low after each experimental condition in both groups.
Renaud *et al*.,^38^ 2014, Canada	CC*	Twenty‐two DSPs (All males, age = 43.5, SD = 13.7) participants in correctional settings, either beginning treatment or involved in pre‐sentencing assessment. All DSP participants had a history of engaging in inappropriate sexual conduct with minors. Forty‐two NDSPs (age = 40.7, SD = 11.5) participants recruited through newspaper advertisements, no criminal record, and no reported sexual interest in children.	The VR system used a head‐mounted display with ocular tracking. Realistic naked humans were depicted according to the Tanner Stages and curated using 3D CGC The intervention consisted of an audio stimuli condition and a VR stimuli condition. In the audio condition, participants encountered scenarios with varying levels of violence and sexual contact with a prepubescent child. The VR portion of the intervention consisted of 5 virtual characters presented for 90 s. Penile plethysmography was used to measure sexual interest.	Primary Outcome: CGC technology yielded significantly better group classification accuracy and discriminant validity for sexual preferences than audio stimuli. Assessing arousal using VR stimuli 54/60 participants were correctly classified as DSP or NDSP, using audio stimuli 44/60 participants were correctly classified. CGC in VR yielded an Area Under the Curve (AUC) of 0.90 (SE = 0.052) while the auditory modality yielded AUC of 0.79 (SE = 0.059)
Hendriks *et al*.,^50^ 2023, The Netherlands	CO*	Ten MHPs (All males = 9, age = 40.4, SD = 10.469) were recruited from either the Forensic Psychiatry Clinic or Long‐Term Intensive care unit of the mental healthcare facility Inforsa. This study did not have a control population. Three therapists (females, age = 32.3, SD 1.528) were also employed by Inforsa and served as a control group.	The VR system used a head‐mounted display. VReedom is a VR‐assisted exposure therapy designed to prepare forensic psychiatry patients for their first authorized leave by exposing them to real‐world scenarios with social, environmental, and feared triggers. The intervention consisted of 6 sessions over a 6‐week program.	Primary Outcome: Preliminary results show patients had low overall stress levels and low variability of stress levels in anticipation of their first authorized leave after VReedom intervention. VR scenarios were effective in eliciting emotional responses which support the goal of exposure‐based therapy. Patients with specific fears (e.g. being alone, crowds) found to benefit most from treatment.
Hubal *et al*.,^34^ 2008, USA	CO	One hundred ninety‐four (n‐194) aggressive prisoners (All male, ages 21–49) with a minimum 18 months left on their sentence were recruited across 3 Maryland correctional institutions during intake into a CBT program. There was no control population.	The VR intervention used virtual embodied conversational agents (ECAs) displayed on a computer screen consisting of short and focused interactions with a virtual character. These stimulated interpersonal verbal interactions with appropriate body language to invoke particular cognitive functions such as risky decision making, impulsivity and sensitivity to penalties. The intervention consisted of 50 sessions across 7 months of a CBT program with patient follow‐up at the 3‐month and 6‐month mark.	Primary Outcome: No significant reduction in risky decision making behavior across the three ECA vignettes after CBT with 12–25% still engaging in risky behavior. No significant improvement in social competency skills of emotional control, information seeking, and negotiation between baseline and post‐treatment. Average number of conversational turns dropped from 2.5 at baseline to 2.4 post‐treatment, could indicate lack of engagement or opportunity. Secondary Outcome: Increase in observed behaviors of engagement, verbalization, and comprehension post‐treatment compared to baseline (*F*_(1,96)_ = 11.08, *P* < 0.001).
McLauchlan & Farley,^42^ 2019, New Zealand	CO*	Six prisoners were selected by the Department of Corrections in New Zealand for participation. There was no control population.	The VR system used a head‐mounted display, 2 controllers, and a tablet. It comprised language lessons identifying car parts and tools in a mechanic's workshop, and completing activities on the tablet. The intervention spanned 10 weeks, and consisted of participants engaging in 40–100 h of both VR and tablet‐medicated activities.	Primary Outcome: All participants showed improved literacy and/or numeracy scores of at least 2 steps on the Literacy and Numeracy for Adults Assessment Tool. Secondary Outcome: Tutors observed higher levels of engagement compared to conventional literacy and numeracy classes. VR facilitated scenarios that allowed for some prisoners to become educators to other prisoners.
Moreno *et al*.,^53^ 2023, Spain	CO	Thirty‐nine IPV offenders (All males, age = 39.18, SD = 11.53) were recruited from the CONTEXTO program: a community psychoeducation program for men convicted of gender‐based violence. Forty‐two general offenders (age = 39.14, SD = 10.98) were recruited from the Service of Management of Penalties and Alternative Measures of Valencia. Forty‐three non‐forensic controls (age = 33.93, SD = 14.10) were recruited from social media. The experiment was conducted in the psychobiology lab at the University of Valencia.	The VR system used a head‐mounted display and also used tablets. Consisted of 1 session split into 2 parts, lasting approximately 60 min total. Consisted of a “compassion eliciting task” where participants viewed the documentary ‘Clouds Over Sidra’, an adaptation of a United Nations documentary. Afterwards, participants could “alleviate suffering” *via* a donation using financial compensation they had received. vmHRV taken as a measure of parasympathetic activity and emotion regulation.	Primary Outcome: Significant difference between groups for vmHRV in Root Mean Square of Successive Differences (RMSSD) during the compassion eliciting task *F*_(2,121)_ = 4.525, *P* = 0.013, *η*p^2^ = 0.070. Post‐hoc analysis reveals lower RMSSD in IPV offenders compared to non‐forensic participants in all components of the compassion eliciting task. This suggests that it may be more difficult for IPV offenders to empathize with the suffering of others. No significant differences in self‐reported emotion and behavior. Highlights use of vmHRV as a sensitive biomarker.
Renaud *et al*.,^36^ 2010, Canada	CO*	Ten SAACs (All males, age = 39.9, SD = 13.6) engaging in treatment or pre‐sentence assessment were recruited from the Royal Ottawa Forensic program, the Centre de psychiatrie le ´gale de Montréal, and the Centre d'E ´tude et de Recherche de l'Université de Montréal. Fifteen controls with no criminal record or sexual interests towards children (age 45.5, SD = 11.0) were recruited from the newspaper.	The full VR system used 2 different modalities. Firstly, a Cave Automatic Virtual Environment system (CAVE) type immersive system where participants wore stereoscopic glasses when viewing dynamic images projected on 3 walls to experience virtual stimuli in 3 dimensions. Secondly, a head‐mounted display presenting virtual stimuli. Sexual arousal was measured throughout the VR intervention using penile plethysmography. Participants were presented 3D animations for five 120 s periods. During these periods they were shown a female adult, female child, male child, male adult, and neutral virtual characters.	Primary Outcome: Virtual characters of validated appearance and age yielded significantly different sexual arousal responses between SAAC offenders and controls (Wilks = 0.489;*F*_(4,20)_ = 5.235; *P* < 0.01). SAAC offenders showed significantly greater arousal, measured using penile plethysmography, to the female child stimuli compared to controls (mean difference = −1.4, *P* < 0.001)
Wijk *et al*.,^35^ 2009, Sweden	CO*	Eight mentally disordered offenders (7M, 1F) from forensic psychiatric care for violent crime. Six of the patients had autistic traits and two had psychotic illnesses. Thirteen staff working with forensic psychiatric patients served as the control population.	The VR system used a computer‐based simulation system. Patients engaged in simulated scenarios where they were presented with a series of decisions related to social and challenging situations, followed by video sequences showing the consequences of their actions.	Primary Outcome: The VR intervention was well received by both patients and staff. Participants rated the interface, visual design and realism of the intervention highly. Participants also indicated they were not stressed using the intervention.
Wit‐De Visser *et al*.,^58^ 2025, The Netherlands	CO	One hundred eight participants (ages 18–65) were recruited from 2 sites Mental Health Center West North Brabant and Fivoor forensic and intensive psychiatry in the Netherlands. To be eligible patients needed to be known with at least one form of antisocial behavior for at least 1 year.	Participants completed the Aggression Catwalk in Virtual Reality (AC‐VR) which consisted of interactions with a virtual character at 4 levels of verbal aggression to assess state mentalization, how individuals perceive and interpret others' behavior and mental states in an interactive context.	Primary Outcome: Latent Class Analysis on AC‐VR identified 5 distinct state‐mentalizing profiles based on the experienced anger, threat, and trust for level of aggression ranging from 1 to 4 (entropy R^2^ = 0.94). The 5 profiles and distributions were medium aroused‐high trust (MA‐HT) 28.9%, medium aroused‐low trust (MA‐LT) 21.1%, low aroused‐high trust (LA‐HT) 18.9%, high aroused‐low trust (HA‐LT) 18.5%, and low aroused‐low trust (LA‐LT) 12.6%. There was no significant relationship between class of trait mentalization as measured by ERART and state mentalization as measured by AC‐VR (Wald = 4.63, *P* = 0.80) which supports the distinction between trait and state mentalizing.
Collins *et al*.,^44^ 2020, New Zealand	CS*	Fifteen male prisoners were recruited from the Otago Correctional Facility in New Zealand with varying levels of education and previous exposure to VR. They were divided into three groups with six, five, and four learners in each. There was no control population.	The VR system used a head‐mounted display. Participants completed 3 sessions and were immersed in a virtual learning environment of a mechanic workshop. At any given time during a session they would be in one of three tasks: a Virtual Mechanic application, a tablet‐based activity, or taking a break, talking, or giving feedback.	Primary Outcome: VR‐based learning was feasible, engaging, and well‐tolerated by incarcerated learners within correctional environments with ethical and regulatory constraints. Researchers observed that participants needed to build trust before feeling comfortable using head‐mounted displays, over‐involvement from support staff interrupted participant focus and ambient noise and peer distractions were frequent and interfered with engagement.
Seinfeld *et al*.,^41^ 2018, Spain	NRCT	Twenty convicted IPV offenders (All males, age = 38.75, SD = 8.52) offenders were recruited *via* the Catalan Justice Department. Nineteen controls (All males, age = 35.95, SD = 10.63) were recruited that had no history of domestic violence.	The VR system used a head‐mounted display as well as a depth sensor to enable body tracking. The VR intervention utilized virtual embodiment as a form of perspective taking to examine its relation to empathy and aggression. Participants were immersed as a virtual female victim in a domestic violence scenario against a male aggressor.	Primary Outcome: IPV offenders were significantly worse at identifying fearful female facial expressions (posterior probability = 0.97) and have more bias towards identifying both male (posterior probability = 0.99) and female (posterior probability = 0.80) fearful expressions as happy rather than fearful. Post‐VR intervention, IPV offenders significantly improved identification of fearful female expressions (posterior probability = 0.99) and decreased response bias towards misattributing fearful expressions as happy in males (posterior probability = 0.96) and females (posterior probability = 0.83).
Seinfeld *et al*.,^54^ 2023, Spain	NRCT	Thirty‐one IPV offenders (All males, age = 41.84, SD = 10.08) with history of aggression against women were recruited *via* the Catalan Justice Department. Nineteen controls (All males, age = 40.58, SD = 10.47) without a history of domestic violence were recruited from the community.	The VR system used a head‐mounted display and a full‐body tracking suit. Participants were immersed in a VR domestic violence scenario from a child's perspective.	Primary Outcome: IPV offenders had a substantial increase in their sensitivity to recognize anger in female faces (posterior probability = 0.956) and bias towards classifying male faces as expressing anger (posterior probability = 0.939). They also had a slight increase in the ability to recognize fear in female faces. IPV offenders had greater Heart Rate Deceleration (HRD) responses to explicit violence (posterior probability = 0.775) while controls had greater HRD responses to implicit violence (posterior probability = 0.912). Secondary Outcome: Both groups rated highly that VR helped them better understand a child's feelings during domestic violence exposure (median score = 6 on Likert scale)
Alshaer,^48^ 2023, Saudi Arabia	PP	Forty‐six participants (age = 23) were recruited from Makkah prison in Saudi Arabia in coordination with Tarahm (a third‐party committee that supports prisoners and their families). There was no control population.	The VR system used a head‐mounted display and controllers. The VR scenario centered around mechanical training in assembling an engine. The study used a within subject's design and participants performed the same VR task using both headsets. Participants also compared the VR‐based training to their traditional training from either the prison or Tarahm.	Primary outcome: VR‐based training had a significantly higher average acceptance rating (1–7) compared to traditional services (VR: 6.601 (SD = 0.629), Traditional: 4.709 (SD = 2.148), *t* = −5.516, df = 46, *P* < 0.001, Cohen's d = −0.805, SE = 0.259). Secondary outcome: comfort and presence mean scores (6.213 to 6.426), dizziness means score (3.298 to 3.596).
Ivarsson *et al*.,^51^ 2023, Sweden	PP*	Fourteen offenders (Age = 29, SD = 8.1) with a history of violent crime and repeated misbehavior before age 15 were recruited from medium and high‐security prisons in Sweden. Most had prior convictions and substance use issues. There was no control population.	The VR system used a head‐mounted display. Revised VRAPT, designed as a form of cognitive behavioral theory and aligning with the General Aggression Model. Combines virtual environments and facilitator‐controlled avatars for real‐time role‐play to help participants practice aggression management. The intervention lasted 8 to 16 weeks (mean = 17.5 weeks, SD = 11.6), with a follow‐up 3 months post intervention.	Primary Outcome: There was a significant reduction in aggression scores (AQ‐RSV) from pre‐treatment to post‐treatment (estimated change = 8.5 points, probability of decrease = 97.45%) and 3‐month follow‐up (estimated change = 9.03 points, probability of decrease 96.74%). Secondary Outcome: There was significant decreased emotion dysregulation (DERS) (post‐treatment estimated change = 18.37, 99.99% probability; follow‐up estimated change = 15.6, 99.89% probability), decreased trait anger (STAXI‐Trait) (post‐treatment estimated change = 4.46, 99.95% probability; follow‐up estimated change = 3.05, 98.92% probability) and decreased anger expression (STAXI‐AX Index) (post‐treatment estimated change = 17.43, >99.99% probability; follow‐up estimated change = 11.18, 99.75% probability)
Sappelli *et al*.,^59^ 2025, The Netherlands	PP*	Twelve participants (All male, age = 36.7, SD = 8.7) were recruited from a high security forensic psychiatry centre and an outpatient forensic psychiatry clinic. All participants had an above‐average or high disposition to react aggressively as measured by a score of 5 or higher on the Social Dysfunction and Aggression Scale‐9. There was no control population.	The VR system used a head‐mounted display. In the VR scenario, participants engaged with various scenes at a café. Participants encountered several different kinds of scenes including an instruction scene, neutral scene, and 2 provocation scenes. In the provocation scenes participants encountered provocations from virtual avatars operated by the researchers including a female avatar and a male bouncer avatar.	Primary Outcome: Observed aggression during VR scenarios as measured by the Social Aggression and Dysfunction Scale‐Virtual Reality Aggression Assessment (SDAS‐VRAA), a modified version of the SDAS‐9, differed significantly across the different scenes (*X* ^2^ *F*_(3)_ = 28.71, *P* < 0.001). Both provocative scenes elicited significantly higher aggression than the neutral and instruction scenes (all *P* < 0.01). The male bouncer provocation scene elicited higher aggression than the female provocation scene (*z* = 2.04, *P* < 0.05).
van Gelder *et al*.,^47^ 2022, The Netherlands	PP	Twenty‐four convicted offenders (All males, age = 23.7, SD = 3.7) from correctional facilities in the Dutch Probation Service There was no control population.	The VR system used a head‐mounted display with two controllers. The intervention utilized virtual embodiment to body swap participants with their “future self” to increase vividness of the future self. Participants first embodied their present self and responded to statements about their behaviors. Next, they embodied their future self (10 years older) and categorized these behaviors as harmful or beneficial. They then returned to their present self and received a ‘future‐self score’ based on their categorizations. Finally, they re‐embodied their future self to offer advice to their present self. Follow‐up was conducted 1‐week post intervention.	Primary Outcome: Interacting with a VR representation of one's future self‐decreased engagement in self‐defeating behavior at 1‐week follow‐up t (20) = −1.57, *P* = 0.07, d = 0.34. There were significant reductions in “Spending more money than intended”: from 71.4% (T1) to 52.4% (T3), *z* = −1.69, *P* = 0.05 and “Drinking alcohol”: from 52.4% (T1) to 28.6% (T3), *z* = −1.97, *P* = 0.02. Vividness of the future self‐increased significantly post intervention (*Δ* = 0.492, SE = 0.196, *P* = 0.01, d = 0.54) and was associated with reduced self‐defeating behavior (*β* = −0.582, *P* = 0.01). There were no significant changes to future self‐concepts such as connectedness, similarity and valence post VR intervention. Secondary Outcome: Participants reported relatively high median ratings on VR experience dimensions (on a 7‐point scale): (Engagement = 4.8; Presence = 4.8; Physical Embodiment = 4.5; Cognitive Embodiment = 4.0). Participants reported no (71%) or very mild levels of (29%) discomfort during the VR experience
Smeijers *et al*.,^45^ 2021, The Netherlands	RCT*	Thirty‐one forensic psychiatric outpatients (All males, age = 36.13, SD = 12.88) with aggression regulation problems. 15 received a CBT‐based intervention and participated in a virtual reality game for aggressive impulse management. 16 received CBT and a control VR game. There was no control population.	The VR system used a head‐mounted display. The intervention Virtual Reality Game for Aggressive Impulse Management (VR‐GAIME) was developed from the motivational modification paradigm. Participants acted as couriers collecting packages. Controls encountered agreeable avatars while the experimental group faced disagreeable avatars to train avoidance behaviors. Both groups also received their usual aggression replacement training. Treatment took place over a course of 12 weeks.	Primary Outcome: Although aggressive behavior reduced over the course of treatment (self‐report: *F*_(1,29.05)_ = −1.84, *P* = 0.076; clinician rated: *F*_(1,25.23)_ = −2.12, *P* = 0.044), the VR intervention was not more successful in reducing anger and aggressive behavior relative to the control condition Wilks' Lambda = 0.312, *F*_(7,20)_ = 0.77, *P* = 0.697. Secondary Outcome: Several patients reported that playing the game gave them insight into their own emotional triggers and awareness that others may not have negative intentions.
Smith *et al*.,^55^ 2023, USA	RCT*	Forty‐four participants (All males, age range = 26–58) were recruited *via* vocational rehabilitation programs from two prisons in Michigan. 28 participants received both their normal employment services as well as VR‐JIT. Sixteen participants only received their normal employment services and served as the control population.	The VR system, Virtual Reality Job Interview Training (VR‐JIT), is a computerized job interview simulator delivered *via* the internet and does not require a head‐mount system. Participants were randomly assigned to receive both their normal employment services and VR‐JIT or only service as usual. There were seven VR job interview training sessions, each session lasted 60 min.	Primary Outcome: Participants who received VR‐JIT group had a significant improvement over those without in their performance‐based job interview skills score (*F*_(1,18)_ = 3.4, *P* = 0.04, *η*p 2 = 0.16). Significant improvements were observed in areas such as “communicates in a positive way” (*F*_(1,18)_ = 10.5, *P* = 0.005, *η* ^2^ = 0.37), “sounds interested” (*F*_(1,18)_ = 4.7, *P* = 0.02, *η* ^2^ = 0.21), training motivation (*F*_(1,19)_ = 4.6, *P* = 0.04, *η* ^2^ = 0.19), and reduced interview anxiety (*F*_(1,19)_ = 5.1, *P* = 0.02, *η* ^2^ = 0.21). At a 6‐month follow‐up 82.1% of participants receiving VR‐JIT obtained jobs *vs*. 68.8% of participants who did not receive VR‐JIT (non‐significant difference, *P* = 0.15) Secondary outcomes: VR‐JIT was highly acceptable (M = 31.1, SD = 3.6, range = 21–35; max = 35) and highly usable (M = 23.6, SD = 0.7; range = 22–24; max = 24)
Smith *et al*.,^60^ 2025, USA	RCT	One hundred and one participants were recruited *via* vocational rehabilitation programs from two prisons in Michigan. Participants were randomized in a 2:1 ratio with 35 completing service as usual and 66 completing both their normal employment services as well as VR‐JIT.	The VR system, Virtual Reality Job Interview Training (VR‐JIT), is a computerized job interview simulator. It is typically delivered *via* the internet but was locally installed and delivered *via* Chromebook laptops due to restrictions on internet access within the prisons. Of the 66 participants assigned to receive VR‐JIT, 58 completed 7–20 60 min interview training sessions and 52 successfully advanced through the recommended difficulty levels from easy to medium to hard.	Primary Outcome: Participants receiving VR‐JIT had higher employment rates at 6 months (82.3% *vs*. 73.5%, *P* = 0.15) and faster time to employment (72.7 days *vs*. 86.0 days, *P* = 0.17) compared to participants receiving service as usual but these findings were non‐significant. After adjusting for covariates VR‐JIT was significantly associated with higher employment rates (OR = 3.76, *P* = 0.032) and faster time to employment (HR = 1.62; *P* = 0.037). Secondary Outcome: Participants receiving VR‐JIT had a moderate improvement in job interview skills (d = 0.60), small increase in motivation to practice job interviewing (d = 0.23), and a small reduction in interview anxiety (d = 0.19) compared to participants receiving only service as usual.
Tuente *et al*.,^43^ 2020, The Netherlands	RCT	One hundred and twenty‐eight participants were recruited from four forensic psychiatric centers in the Netherlands. All participants had a history of aggression and/or current clinical problems with aggression. 64 of these participants (Age = 39.4, SD = 10.6) received virtual reality aggression prevention training. The other 64 participants (Age = 38.0, SD = 10.0) received their usual treatment and served as the control population.	The VR system used a head‐mounted display. The intervention, virtual reality aggression prevention training (VRAPT), was based on the Social Information Processing model and combines role play with therapist‐controlled avatars and physiological markers such as heart rate (HR) and galvanic skin response (GSR). Consisted of 16 one‐hour individual treatment sessions, twice a week. Assessments were done at baseline, post‐treatment and at 3‐month follow‐up.	Primary Outcome: VRAPT was not effective in reducing aggressive behavior based on staff rating (post‐treatment: *β* = 0.15, *t* = 0.27, *P* = 0.79) and self‐reports (post‐treatment: *β* = 0.18, *t* = 0.06, *P* = 0.95; 3‐month follow‐up: *β* = −1.70, *t* = −1.10, *P* = 0.27) VRAPT led to temporary, self‐reported improvements in anger control and impulsivity that were not maintained at 3‐month follow‐up. 76.3% of participants believed VRAPT was a valuable addition to their treatment. Secondary Outcome: Igroup Presence Questionnaire (IPQ) 0–6: Spatial presence: Mean = 3.12 (SD = 1.1), Involvement: Mean = 2.5 (SD = 1.6), Realness: Mean = 2.18 (SD = 1.3)
Bacon *et al*.,^37^ 2012, Australia	MM	Two forensic mental health patients (1M, 1F, age = 35.5) at risk of obesity due to psychotropic medication were recruited from Thomas Embling forensic mental health hospital in Australia. Although more participants had participated, only 2 completed the study and gave adequate informed consent. There was no control population.	Participants engaged in Wii Sports games on the Nintendo Wii Fit as a form of physical activity. The Wii Fit was available to patients for 8 weeks and patients were encouraged by researchers to play. Activity was monitored by staff and tracked by an accelerometer. Participants completed a 30‐min interview after the 8‐week intervention.	Primary Outcomes: Wii Fit helped forensic mental health patients at risk of obesity increase engagement in physical activity (patient 1 mean weekly time spent 61.63 mins for 8 weeks; patient 2 mean weekly time spent 114 mins for 5 weeks) and to positively change their attitudes towards it. Patient 1 lost 3.4kg over 8 weeks, patient 2 lost 1kg over 5 weeks.
Claborn *et al*.,^57^ 2024, USA	MM*	Twenty incarcerated individuals (10M, age = 32.47, SD = 8.369) with opioid use disorder were recruited from a county jail. There was no control population.	The VR system used a head‐mounted display, 2 motion controllers, and 2 external motion sensors. Participants engaged with an immersive 3D VR environment in a green meadow where they could move freely. Data was collected continuously during VR exposure and interviews, but session durations were not specified.	Primary Outcome: Significant decrease in heart rate (HR) during VR exposure [b = −3.14, t (18) = −3.85, *P* < 0.01]. Secondary Outcomes: Qualitatively, high acceptability and perceived utility of VR for mental health and substance use interventions, community reentry skills training, and communication and conflict resolution skills.
Haneveld *et al*.,^61^ 2025, The Netherlands	MM	Six forensic psychiatry inpatients (All males, age = 33.4, SD = 9.3) treated for aggression regulation problems were recruited from Dutch inpatient clinics. Five healthcare professionals (1M, 4F, age = 31.2, SD = 7.1) were recruited from Dutch inpatient clinics.	The VR system used a head‐mounted display. Patients engaged with VR biofeedback game ‘DEEP’, navigated an immersive underwater VR environment by using their diaphragmatic breathing with visual biofeedback from changing corals and plants. The experiment was conducted between May 2022 and October 2022.	Primary Outcome: Effectiveness of DEEP varied across participants. Of the 6 patients, only 2 patients had significant reductions in stress over time (*β* = −0.014, *P* = 0.007; Stress: *β* = −0.029, *P* < 0.001). Qualitatively, 5 out of 6 patients endorsed DEEP having a positive effect including feeling “relaxed” and “calm after sessions as well as helpful to improve awareness of their breathing. Health care professionals similarly reported observing a relaxing effect of DEEP in their patients but note that most patients were not yet able to incorporate what they learned in DEEP into practice.
Hedstrom *et al*.,^49^ 2023, Sweden	MM*	Ten forensic psychiatry inpatients (age = 35.8, SD = 9.6) with a history or indication of current paranoid ideation were recruited from a high security forensic psychiatric clinic in Sweden. There was no control population.	The VR system used a head‐mounted display. Participants engaged with neutral VR scenarios, in a supermarket and seated on a bus, intended to be relatable to most individuals. Scenarios lasted 5 min each. Total intervention duration was 1.5 to 4 h completed over 1 or 2 days.	Primary Outcome: State paranoia (SSPS) was slightly higher in the supermarket scenario (Mean = 15.2, SD = 9.6) than bus scenario (Mean = 12.7, SD = 5.0). Perceived hostility in supermarket scenario (Mean = 23.3, SD = 27.2), perceived hostility in bus scenario (9.3, SD = 11.5). Paranoia in the bus scenario correlated with social anxiety (*r* = 0.64, *P* = 0.05), ideas of social reference (*r* = 0.77, *P* < 0.01), ideas of persecution (*r* = 0.66, *P* = 0.04), and total paranoid thoughts (*r* = 0.81, *P* < 0.01). Perceived hostility in the bus scenario correlated with ideas of reference (*r* = 0.69, *P* = 0.03) and total paranoid thoughts (*r* = 0.67, *P* = 0.04). No significant correlations in the supermarket scenario. VR immersion and presence (IPQ) General presence (M = 5.1, SD = 1.0), Spatial presence (M = 4.0, SD = 0.9), Involvement (M = 3.4, SD = 1.1), Realness (M = 2.9, SD = 1.1) Secondary outcomes: Qualitatively patient and clinicians endorse VR enabling collaborative assessment. Patient engagement was mixed and may have been affected by factors such as familiarity with technology, differences in cognition and motivation, and level of psychotic symptom severity.
van Rijn *et al*.,^39^ 2017, UK	MM	Four repeat offenders (age = 33.8, SD=NR) were recruited from a Category B therapeutic community prison in the UK. There was no control population.	The system was run on a laptop. Participants utilized “ProReal”, an avatar‐based therapeutic program where individuals can create a visual representation of their world or specific situation and then explain it to others. The Intervention lasted 7 group sessions over 3 months (May to July 2014) with each session lasting 90 min.	Primary Outcome: Participants had a non‐significant reduction in psychological distress following intervention (mean reduction on CORE‐10 of 3.7 points, Cohen's d = 0.87, *t* = 1.7, *P* = 0.19). Secondary Outcomes: Improved self‐expression, empathy, interpersonal reflection, and interpersonal relationships between peers and counselor were noted qualitatively.
Woicik *et al*.,^56^ 2023, The Netherlands	MM*	Seventeen participants (All males, age = 32, SD = 8.4) were recruited from a Penitentiary Institution in the Netherlands. Participants had been convicted for a variety of crimes such as manslaughter, property crimes, homicide, etc. Of the 17 participants, 10 completed all 16 sessions. Four therapists were also included in the qualitative evaluation as a control population.	The VR system used a head‐mounted display. The intervention, virtual reality aggression prevention training (VRAPT), was based on the Social Information Processing model and combines role play with therapist‐controlled avatars and physiological markers such as heart rate (HR) and galvanic skin response (GSR). Consisted of 16 one‐hour individual treatment sessions, twice a week. Assessments were done at baseline, post‐treatment and at 2‐month follow‐up.	Primary Outcome: Self‐reported measures of aggression, anger, provocation and emotion regulation decreased with small to medium effect sizes and maintained at follow‐up. Observational measurements showed a slight decrease in physical aggression at post‐treatment, with a small effect size but this decrease was not maintained at follow‐up. Qualitatively, participants reported having learned to respond more adequately to aggressive behavior, using different coping strategies and gaining insight into their own and others' emotional states. Secondary Outcome: Participants and therapists rated average satisfaction score of the intervention as 9.2 out of 10 (SD = 0.3). VR Presence measures (1–7 scale): Spatial presence: 4.0 (SD = 0.6), General presence: 4.6 (SD = 2.1), Involvement: 4.2 (SD = 0.9), Experienced realism: 3.6 (SD = 1.1) 4 participants reported on cybersickness (3 dizziness, 1 described “car‐sickness”).
González Moraga *et al*.,^62^ 2024, Sweden	QS	Seven forensic psychiatry patients (6M, 1F, age = 36) were recruited from a high security forensic psychiatric clinic in Sweden. There was no control population.	Participants previously completed virtual reality aggression prevention training (VRAPT) treatment within a quantitative pilot study.^51^	Primary outcome: There were 6 main categories that emerged from manifest content analysis: therapeutic process, VRAPT method, VR technology, previous treatment experiences, challenges to treatment of aggression, and unexpected experiences. Participants generally reported that VR‐assisted roleplay was a positive experience and helped enhance emotional awareness, communication skills, and managing aggression, though experiences varied across participants. Challenges reported by participants included variable motivation for completing repetitive tasks, technological limitations affecting immersion, and transference of skills into daily life.
Haneveld *et al*.,^52^ 2023, The Netherlands	QS	Thirteen offenders with criminal behavior related to psychiatric conditions (11M, 2F) were recruited from inpatient forensic psychiatric clinics in the Netherlands. Twenty‐four healthcare providers directly involved with the patients (7M, 14F, 3O) were recruited, including psychologists, coordinating therapists, team managers, forensic nurses, and socio‐therapists.	The VR system used a head‐mounted display. Patients engaged with VR biofeedback game ‘DEEP’, navigated a calm underwater VR environment by controlling their breathing, with visual biofeedback from changing corals and plants. Data was collected between November 2021 and April 2022. Focus groups lasted around 60 min (range: 43–71 min), while patient interviews lasted 20 min (range: 15–27 min).	Primary Outcome: 10 patients and 4 providers reported DEEP may help users to reduce stress and enhance relaxation. 13 providers emphasized DEEP as a useful intervention to teach patients diaphragmatic breathing techniques while 4 patients note focusing on their breathing was helpful for relaxation. 18 providers and 9 patients described DEEP as fun and immersive. 11 providers reported it was accessible and requires few cognitive skills. 5 providers expressed concern using DEEP with patients that have psychotic vulnerability.
Ivarsson *et al*.,^63^ 2025, Sweden	QS*	Thirteen offenders (All males) were recruited from a previous VR pilot study in the Swedish Prison and Probation Service (SPPS). There was no control population.	Participants previously completed virtual reality aggression prevention training (VRAPT) treatment within a quantitative pilot study.^51^	Primary Outcome: VRAPT was found to be a feasible intervention in Swedish prisons. Overall participants found that VRAPT was realistic and engaging, often more so than traditional role‐play. Most participants felt that VRAPT was helpful for skills training, particularly for emotion recognition and de‐escalation. Participants praised the therapists and noted the therapeutic alliance as important for the positive impact of VRAPT.
Kouijzer *et al*.,^64^ 2024, The Netherlands	QS	Eight patients (All males) were recruited from two Dutch mental healthcare organizations Transfore and De Waag. Ten healthcare providers (2M, 8F) working in forensic mental healthcare organizations were recruited. Consisted of 6 GZ psychologists, 2 forensic nurses, 1 occupational therapist, 1 forensic remedial educationalist.	The VR system used a head‐mounted display, 2 controllers, and noise canceling headphones. Patients engaged with the VR intervention ‘Triggers and Helpers’. Participants were immersed in various virtual environments (e.g. supermarket, shopping street, home environment, etc.) and conducted role‐play with virtual characters portrayed by the healthcare provider.	Primary Outcome: Participants were broadly positive in their first impressions of VR finding it to be generably enjoyable. Areas for improvement that were identified included improving realism of VR and reducing physical discomfort while in VR. Providers generally liked VR and its customizability but wanted more support for its implementation.
Mason *et al*.,^46^ 2022, UK	QS*	Five male hospitalized forensic psychiatry patients were recruited. Ten staff (6M) consisting of nursing, OT, psychology, psychiatry were recruited from a medium‐secure forensic hospital in the UK (Birmingham and Solihull Mental Health Foundation Trust).	Non‐immersive setup, the specific VR system was not specified. Participants utilized “ProReal”, an avatar‐based therapeutic program where individuals can create a visual representation of their world or specific situation and then explain it to others. The intervention consisted of 84 sessions, lasting 30–90 min each (February 21, 2019 – March 9, 2020) while evaluation interviews lasted 10 to 42 min (mean = 22 min).	Primary Outcome: Demonstrated feasibility and acceptability of avatar‐based software (ProReal) to enhance communication, emotional expression, mentalisation, and reflective practice.
Sivermo *et al*.,^65^ 2025, Sweden	QS*	Seven patients (6M 1F, age = 36) with an established pattern of violence and current issues with reactive aggression were recruited from a maximum‐security forensic psychiatric clinic.	The VR system used a head‐mounted display. Revised VRAPT, designed as a form of cognitive behavioral theory and aligning with the General Aggression Model. Combines virtual environments and facilitator‐controlled avatars for real‐time role‐play to help participants practice aggression management and self‐management of physiological reactions (e.g. measured by heart rate variations and skin conductance). Conducted over 16 sessions.	Primary Outcome: There were 3 manifest content categories identified relating to treatment content: skills‐training, tailoring of the intervention, and self‐awareness. The collaborative nature of VRAPT and the therapeutic alliance between patients and therapists was recognized as being especially important to facilitate role‐play. Secondary Outcome: Patient goals were largely around strategies for managing aggression while therapists focused more on identifying triggers of aggression.

* A pilot/feasibility/preliminary/exploratory study.

CBT, cognitive behavioral therapy; CC, Case–Control; CGC, computer‐generated characters; CO, Cohort‐Observational; CS, Case Study; CSS, Case Series Study; DSP, deviant sexual preference; F, female; IPV, intimate partner violence; M, male; MHP, mental health patient; MM, Mixed‐Methods; NDSP, non‐deviant sexual preferences; NOC, non‐offender controls; NRCT, Non‐Randomized Control Trial; O, other; PP, Pre‐Post Study without Control; QS, Qualitative Study; RCT, Randomized Control Trial; SAAC, sexual aggressors against children; SOC, sexual offenders against children; vmHRV, vagally mediated heart rate variability; VR, virtual reality.

### Characteristics of VR interventions

The included studies covered a diverse range of VR interventions, utilizing varying modalities, the majority of which were immersive (n = 25). Immersive VR interventions employed head‐mounted displays (HMDs) to interface participants with realistic virtual environments. One study additionally utilized a CAVE‐type immersive system. Several interventions (n = 4) paired HMDs with motion‐tracking devices to better enable perspective embodying or simulating risk scenarios.^40^, ^53^ In contrast, non‐immersive VR setups (n = 7) such as avatar‐based or scenario‐based simulations enabled participants to explore virtual scenarios on standard computer screens. Interventions ranged in length from single sessions to multi‐week or even multi‐month programs.^34^, ^36^, ^42^, ^50^, ^53^

A variety of VR interventions were delivered in the included studies. Exposure‐based designs were utilized for addressing high‐risk behaviors, including placing sexual offenders against children in virtual scenarios involving child characters,^38^, ^40^ while intimate partner violence (IPV) offenders were confronted with domestic conflict simulations or perspective‐taking experiences as a virtual victim.^41^, ^54^ Other interventions targeted broader clinical or rehabilitative goals, such as vocational readiness,^55^, ^60^ virtual role‐play scenarios for aggression management,^43^, ^45^, ^62^, ^63^, ^65^ and virtual vignettes to prompt decision‐making and self‐reflection.^34^, ^35^

### Application of VR for assessments of symptoms and risk

Eight studies explored the use of VR‐based interventions for the purpose of assessment.^34^, ^35^, ^36^, ^38^, ^40^, ^49^, ^50^, ^58^ Among these studies (n = 8), three of them use VR for the assessment of paraphilia, particularly pedophilic disorders.^36^, ^38^, ^40^ Notably, when penile plethysmography was used to assess sexual arousal, computer‐generated virtual child characters were successful in eliciting arousal from individuals who had deviant sexual preferences.^36^, ^38^, ^40^ VR was also more accurate compared to traditional audio stimuli at classifying pedophilic preferences, with 90% of participants correctly classified as having either deviant sexual preferences or non‐deviant sexual preferences compared to only 73% when using audio stimuli.^38^

Aside from paraphilias, simulation style interventions were also utilized for risk assessment.^34^, ^35^, ^50^, ^59^ In these studies (n = 4), participants were exposed to scenarios one could encounter in everyday life allowing for assessors to observe how they may react in the community and for individuals to practice and receive feedback on conflict‐resolution strategies. For instance, VR successfully elicited aggression following virtual encounters with provoking avatars in a café scene.^59^ In their study, Hendriks *et al*.^50^ reported that forensic patients had low levels of stress in anticipation of their first authorized leave following VR intervention. On the other hand, virtual simulations were observed to be insufficient in assessing risky decision making behavior and the effectiveness of cognitive behavioral therapy among high‐risk prisoners.^34^ Similarly, Hedström *et al*.^49^ reported limited reliability of VR as a standalone assessment tool for paranoid ideation in forensic psychiatric patients. One study assessed individuals with antisocial behaviors, in particular using VR to measure mentalizing abilities during simulated interactions and finding that individuals broadly fell within one of five profiles.^58^

### Application of VR for aggression and violence management

Findings were somewhat mixed for the management of aggressive and violent behaviors among the included studies. Six studies utilized different versions of Virtual Reality Aggression Prevention Therapy (VRAPT) for managing aggression.^43^, ^51^, ^56^, ^62^, ^63^, ^65^, ^66^ A multicenter RCT study reported positive effects of VRAPT on self‐reported anger control and impulsivity, but no significant effect on staff‐reported aggression or long‐term effects when measured at 3‐month follow‐up among forensic patients.^43^ Conversely, a pilot study of VRAPT in a prison population showed improvements in self‐reported measures of aggression but with only small to medium effect sizes. In this pilot study, participants reported learning how to use coping strategies and responding more adequately to aggressive behaviors.^56^ Another pilot study using the revised version of VRAPT (which utilizes CBT techniques) showed significant reduction in aggression and emotional dysregulation both post‐treatment and at 3‐month follow‐up.^51^ Three studies investigating VRAPT conducted qualitative interviews with participants, two of which utilized patients that had previously participated in VRAPT from another pilot study.^62^, ^63^, ^65^ Participants were generally positive with respect to their experience, reporting that VR‐assisted roleplay was helpful in developing skills such as emotional awareness and recognition, communication, and de‐escalation of aggression. Virtual Reality Game for Aggressive Impulse Management (VR‐GAIME) was another intervention developed for aggression management based on the motivational modification paradigm;^67^ however, studies found no significant difference between VR‐GAIME and the control group.^45^ Nonetheless, participants from both VRAPT and VR‐GAIME expressed that the interventions helped them develop better insight into their own and others behaviors.^45^, ^56^

On another note, three studies investigating the application of VR interventions showed promise in modifying maladaptive responses and enhancing empathy among intimate partner violence (IPV) offenders.^41^, ^53^, ^54^ Participants were immersed in domestic violence scenarios from either the victim's or a child's perspective, which led to improved recognition of fearful and angry female facial expressions and reduction in biased or misattributed interpretations of male facial expressions.^41^, ^54^ Participants also reported that VR helped them empathize with children’ s feelings during domestic violence exposure. In a separate study, offenders were found to exhibit lower vagally mediated heart rate variability (vmHRV) during a compassion‐eliciting VR task; a biomarker associated with maladaptive emotion regulation which also serves as a measurable outcome to track therapeutic change over time.^53^

### Application of VR for stress management and relaxation

A subset of interventions (n = 3) targeted stress management and relaxation. These studies utilized VR to immerse participants within calming natural environments leading to decreased heart rate and many participants reporting that it provided them with a feeling of calm and escape.^52^, ^57^, ^61^ ‘DEEP’ was a VR intervention that incorporated real‐time biofeedback on breathing through changes to a virtual underwater environment.^52^, ^61^ While only two of six participants were found to have significant long‐term reductions in stress after DEEP, the majority of participants commented on DEEP having a positive effect.^52^, ^61^ In particular, it provided short‐term relaxation and calm as well as helped participants become more cognizant of their breathing with many healthcare providers noting that DEEP was a useful adjunct to existing breathing exercises.

### VR for skill development and community reintegration

A number of VR interventions were focused on positive behavioral changes and community reintegration.^34^, ^37^, ^47^, ^50^ ‘VReedom’ combines VR with exposure therapy in order to prepare individuals for potential social and environmental triggers as they reintegrate into society.^50^ The results showed lower stress in anticipation of first authorized leave, especially in patients with specific fears such as being alone or in crowds. VR was also used to deliver interventions to improve insight and reflection, utilizing virtual embodiment to facilitate interaction between convicted offenders and virtual avatars representing their future self.^47^ At 1‐week follow‐up, offenders had a reduction in self‐defeating behaviors, such as ‘spending more money than intended’ or ‘drinking alcohol’, which was associated with increased vividness of one's future self. Lastly, engaging with simulated exercise activities increased overall physical activity and had a positive effect on patient attitudes towards exercise.^37^

Another focus of VR intervention was to enhance education and skill development within forensic and correctional populations in anticipation of returning to the community. Studies found that participants engaging in VR‐based classes made significant improvements to their literacy and numeracy scores and both tutors and researchers noted that prisoners were more engaged with virtual learning environments compared to traditional classrooms.^42^, ^44^ Virtual Reality Job Interview Training (VR‐JIT) is a virtual job simulation style intervention designed to help participants rehearse answers to common questions under realistic interview conditions. Participants reported improvements to their confidence and communication post intervention along with a significant improvement to overall interview performance scores.^55^ Participants receiving VR‐JIT were also found to have 276% higher odds of employment and a 62% higher rate of obtaining employment at 6‐month follow‐up.^60^

### Patient and staff perspectives on VR

VR was reported to be highly engaging as an intervention across the included studies. Therapists described participants as ‘curious and motivated’ to use VR interventions and more engaged when compared to their non‐VR counterparts.^42^, ^48^, ^50^ Both immersive and non‐immersive VR interventions were found to be highly accepted among patients.^35^, ^39^, ^46^, ^55^, ^64^ Similarly, healthcare workers found VR interventions to be highly engaging and acceptable.^35^, ^56^, ^64^ Patients and staff described VR as a valuable addition to treatments and perceived utility in VR for mental health, substance use interventions, community reentry skills training, and communication and conflict resolution skills.^43^, ^46^, ^57^, ^63^ Several healthcare practitioners further highlight the flexibility of VR as beneficial to patients for personalizing therapies and promoting patient engagement and motivation, particularly for individuals with low cognitive skills and poor emotional regulation.^50^, ^52^ While participants were generally positive of their experience with role‐play in VR, several highlighted the therapeutic alliance as essential for facilitating role‐play.^63^, ^65^

### Challenges and barriers to VR implementation

Several barriers to the use of VR were noted in the included studies. Four studies reported cybersickness (a condition characterized by symptoms like nausea, vomiting, dizziness, visual disturbances, and headaches) as a barrier, particularly among first‐time users of VR.^47^, ^49^, ^52^, ^57^ Notably, two studies further reported one patient each dropping out of their study due to the severity of their cybersickness symptoms.^45^, ^56^

Another barrier to VR technologies is the complexity of both hardware and software. In terms of software, Renaud *et al*.^36^ described the difficulty of designing virtual characters, especially when required to fit specific age restrictions and meet a degree of realism. Unrealistic characters and environments were noted to negatively impact immersion.^64^ Other studies have also reported various complications of VR software and hardware, including technical issues with software, limited access to Internet, difficulty navigating heavy equipment, wires impeding comfortable movement of participants, limited technological literacy, and poor confidence to engage with VR.^46^, ^49^, ^50^, ^52^, ^56^, ^58^

On a different note, Wijk *et al*.^35^ noted that the burden of resources needed to implement VR within forensic and correctional settings can constitute as a barrier. For example, significant resources might be associated with the cost of VR software and hardware, training therapists, hiring staff for technical assistance, and creation of scenarios.^42^ Lastly, the forensic‐correctional patient population may find interacting with VR particularly challenging. For instance, Hedström *et al*.^49^ noted that some of their patients may have had difficulty understanding VR interventions due to their psychotic symptoms. Also, VR interventions may not be appropriate for individuals with negative symptoms or significant history of trauma and/or PTSD as some clinical deterioration was observed among these study participants.^34^, ^37^, ^51^, ^54^, ^57^, ^64^

### Assessment of study quality

Of the included studies, seven studies were rated as high quality (greater than 75% of criteria met), 17 studies were rated as moderate quality (40–75% of criteria met), three studies were rated as low quality (less than 40% of criteria met), and five studies were rated moderate‐high (mixed methods, quantitative‐qualitative). A significant number of studies had limited sample sizes, with n = 23 studies having less than 25 total non‐control analyzed participants. See supplementary documents (Tables S2 and S3) for further breakdown of the quality assessment scores.

## Discussion

This systematic review summarizes the current literature on the applications of VR in forensic‐correctional settings. We highlight several important findings, including applications relating to patients' assessment and treatment. We also identify key barriers to the successful implementation of VR in clinical practice in forensic‐correctional settings and make relevant recommendations. Major findings are discussed in subsequent sections below.

### Findings on the use of VR for assessment

An important use for VR among forensic‐correctional populations is in assessments. VR was utilized to assess risk and relevant symptoms (e.g. paranoid ideation), aiding clinicians in developing accurate risk profiles and strategies to effectively mitigate risk triggers and promote community reintegration.^35^, ^49^, ^50^ VR was especially valuable for assessing high‐risk populations, such as sexual offenders against children, and performed better than traditional methods at assessing deviant sexual interests as well as predicting offenders' ability to cope in situations outside of correctional facilities.^36^, ^38^ In high‐risk populations, VR allowed assessments with virtual scenarios and stimuli that would otherwise be ethically challenging to implement.^68^ Such assessments provide valuable information on recidivism and community risk in special offender populations without endangering the general public. For example, patients with pedophilic disorder can have their behavior monitored for adherence to treatment and utilization of coping skills and strategies using specific virtual situations for predicting future risk.^40^ On a broader scale, it is tenable to suggest that VR‐based assessments in forensic‐correctional settings will extend into other areas of assessments currently available in general psychiatric practice, including the assessments of cognition, severity of hallucinations, delusional thought content, stress, maladaptive self‐blame in depressive patients, social anxiety and specific phobias among others.^69^, ^70^, ^71^, ^72^, ^73^

### VR for management of aggression and violence

Violence constitutes a major concern among forensic‐correctional populations, with offenders, patients and staff alike at risk of experiencing violence.^74^, ^75^ VR‐based interventions such as VRAPT demonstrated success at modifying patients' aggression and emotional dysregulation, particularly when VR is used to deliver established therapeutic principles such as CBT and even more so among difficult populations.^43^, ^45^, ^51^, ^56^, ^62^, ^63^, ^65^ Participants were able to practice coping strategies for anger management and develop better insight into their own and others' behaviors when exposed to VR interventions.

### Stress management and relaxation

Immersing participants in tranquil, natural environments was effective for reducing stress and helping patients engage in stress management techniques such as deep breathing.^52^, ^57^, ^61^ These findings are consistent with previous research among highly stressed adults and showed beneficial restorative and coping effects that are crucial for individuals with restrictions of their natural physical environment.^76^, ^77^ Thus, VR may provide an alternative avenue of stress management for individuals within forensic‐correctional settings, and can be beneficial for enhancing resilience to stress similar to programs designed for other regimented populations.^78^

### Skill development and community reintegration

A number of the included studies showed positive impacts of VR interventions on the development of skills needed to support community reintegration and tenure, especially among individuals in forensic‐correctional settings with increased risk of institutionalization.^49^ Specifically, VR was effective in delivering simulated psychosocial skill training and interviews to improve confidence, communication skills and performance during vocational interviews leading to improved employment outcomes.^55^, ^60^ Virtual classrooms allowed individuals in incarceration to effectively learn relevant skills, such as literacy and numeracy, for vocational readiness and reintegration into the community.^42^, ^44^ On a different note, simulative technologies helped to gamify training to improve engagement with physical activity and wellbeing,^37^ similar to the applications of VR in other populations to promote healthy behaviors, such as exercise and eating a balanced diet.^79^, ^80^ Such application of VR is particularly relevant in forensic‐correctional settings to promote wellbeing and curb unemployment, which is a known risk factor for re‐offending.^81^, ^82^, ^83^, ^84^ The superior effectiveness of VR training programs for social skill development compared to non‐VR has been demonstrated in meta‐analytic review, with a superiority that is nearly three‐fourths of the standard deviation of mean.^85^, ^86^

### VR integrated with biomarkers and biofeedback measurements

An underutilized facet of VR is the ability to incorporate physiological measures and responses to provide real‐time feedback as an adjunct to interventions in a process called biofeedback.^15^ For example, VR was effectively augmented with penile plethysmograph testing to provide a more robust physiological assessment of arousal in sexual offenders.^36^, ^38^ Similarly, vmHRV was utilized to track parasympathetic activity as a biomarker for emotion regulation.^53^ Biofeedback can be integrated to augment the user's virtual world with real‐time biological measures thereby increasing participants' awareness of their physiological processes beyond one's normal senses, such as monitoring one's own breathing in the stress management intervention ‘DEEP’.^52^, ^61^ In VRAPT, biofeedback was used to take real‐time measures of heart rate and galvanic skin response as physical measures of arousal in a simulated intervention for aggression.^43^, ^56^, ^62^, ^63^, ^65^ Overall, these innovative applications of VR allowed the collection of rich physio‐biological data to support objective and quantifiable measurement of risk in forensic‐correctional populations.

### Applications VR for healthcare Workers

Only a subset of the included studies included results pertaining to forensic‐correctional healthcare workers, which mainly highlighted VR as an avenue to further patient‐provider collaboration through building patient engagement and rapport.^35^, ^46^, ^50^, ^52^, ^56^, ^61^, ^64^

### Challenges and barriers to VR implementation

A number of challenges and barriers to the implementation of VR interventions were highlighted. These include cybersickness, the knowledge and technical burden on both patients and staff to operate VR, the demand for resources, and underlying concerns around how VR may impact specific forensic‐correctional populations (such as in patients with negative symptoms or significant trauma/PTSD). Some studies commented on the risk‐averse nature of forensic‐correctional settings and how VR hardware and certain VR‐based program's need for internet access may introduce additional risk.^42^, ^44^ Certain VR interventions may also require quiet spaces for clinical benefit, as such, patients who engage with VR in noisy, disruptive environments may receive less benefit. Claborn *et al*.^57^ further highlight the compounding interpersonal difficulties of VR usage in prisons as it may yield jealousy and confrontation among other prisoners.

### Limitations

Several limitations were identified in this review, including limited sample size in the included studies, thereby reducing the statistical power of the results.^37^, ^39^, ^40^, ^42^, ^45^, ^47^, ^53^, ^54^, ^57^, ^59^, ^64^, ^65^ Similarly, many of the included studies were pilot, feasibility, or exploratory in nature and only three were randomized controlled trials. Another limitation is the heterogeneity in the measures and interventions utilized across the included studies. The lack of standardized measures across studies makes it difficult to draw comparisons and conclusions between studies. In a similar vein, reporting on relevant measures of VR was not consistent across all studies. For instance, many studies do not comment on the immersiveness and side effects of VR. These limitations may impact the translation and generalizability of results, supporting the need for future research.

### Study implications and recommendations

This review highlighted many applications of VR in forensic‐correctional psychiatry, ranging from assessment, emotion regulation, skill development, community reintegration, and improvement of patient engagement. Notwithstanding, there is evidence to suggest that the potential benefits of VR in forensic‐correctional settings are yet to be fully tapped. In clinical practice, VR can be integrated into the assessments and management of patients in forensic‐correctional settings while leveraging its unique advantages. For example, VR can allow for precise control of user's exposure to stimuli within an ecologically valid, safe, and virtual environment, allowing for the monitoring of physiological and behavioral responses. This control underscores the capacity to adapt VR interventions for brief, targeted exposures or extended therapeutic courses depending on participants' risk profiles. This is especially important for tailoring interventions to individual needs to allow for better outcomes when targeting lower recidivism rates.^87^ VR can also be used to deliver a structured and nuanced assessment of risk factors, thereby producing accurate risk assessments to inform personalized treatment plans and decision making. It is important to highlight the unique application of VR to simulate exposures necessary for assessment and treatment that would otherwise be limited and fraught with ethical concerns in the real world. This is particularly relevant in sexual offenders where it is imperative to balance the best interests of the patient with public safety.^68^ Furthermore, VR allows for the innovative utilization of biomarkers during assessment and treatment of sexual offenders.

Engagement with VR interventions was high in this review, making it a viable interventional modality among forensic‐correctional populations, who are historically known to have poor participation in treatment.^88^, ^89^, ^90^ In difficult patients, the gamified attribute of VR can be leveraged to improve engagement.^19^

While current research on VR within forensic‐correctional settings has mainly focused on the patients, much of the utility of VR within other sectors of healthcare has been for the training of providers. Hence, it is important to extend the application of VR into training healthcare professionals.^3^, ^91^, ^92^, ^93^ In the same vein, VR interventions can be used to recreate traumatic scenarios for providers to practice therapeutic skills and deal with vicarious trauma which has been reported among healthcare workers working in forensic‐correctional settings.^94^ This is especially important given the identified vulnerability to traumatic exposure and disproportionate mental health burden experienced by healthcare workers within the forensic‐correctional psychiatric field.^95^

Lastly, the big challenge with VR is implementation. For successful implementation, there is a need for active engagement of hospital administrators and other stakeholders to allow adequate allocation of funds to set up the VR technology, develop new software, and create virtual environments and train staff.^10^, ^19^, ^50^ To support its implementation, uptake and evidence‐based practice, future studies are needed to inform guidelines and address the limitations identified in current literature.^96^

## Conclusion

In conclusion, this systematic review highlights the evidence base for the applications of VR in forensic‐correctional psychiatry. Findings suggest promising beneficial effects of VR on accurate risk assessment and better clinical outcomes. VR interventions described in the studies in this review reveal a wide flexibility in both design and applications to support assessment of high‐risk patients, empathy training, skill transfer for job readiness, management of aggression, and collection of biophysiological data on difficult or special populations of offenders (e.g. pedophilic offenders). In spite of the promising results, the application of VR in forensic and correctional psychiatry is yet to be fully tapped. Future high‐quality studies are needed to replicate findings in the existing literature, refine VR's implementation, standardize its use and promote evidence‐based guidelines. As VR and other technologies continue to advance, its capacity to reshape mental health care, especially in forensic and correctional settings cannot be overemphasized. This review represents an important step towards pushing existing boundaries in the application of VR.

## Author contributions

M.Y.W., E.Y., M.M.K., J.M.B., G.A.C., and A.T.O. were involved in conceptualizing the research idea. M.Y.W., R.T., E.Y., M.M., S.T., and A.T.O. were vital in the data collection and curation. M.Y.W., E.Y., R.T., M.M., S.T., M.M.K., J.M.B., G.A.C., and A.T.O. were involved in the data analysis process, interpretation, and presentation. M.Y.W., E.Y., R.T., M.M., S.T., and A.T.O. drafted the initial manuscript, and all authors provided substantial intellectual contributions in the subsequent revisions. M.Y.W., E.Y., and A.T.O. were involved in the visualization of the current manuscript. A.T.O. supervised the various stages of the project. All authors gave final approval of the version of the manuscript to be published and agreed to be accountable for all aspects of the work.

## Disclosure statement

The authors have no conflicts of interest to declare.

## Supporting information


**Table S1a.** Search strategy.
**Table S1b.** Record of Searches.
**Table S2.** Quality assessment with NIH quality assessment tool.
**Table S3.** Quality assessment with CASP.

## Data Availability

The data that support the findings of this study are in the manuscript and/or in the supporting documents file. Additionally, the corresponding author can be contacted for further information.
