# Supplementary material for: The use of virtual reality in forensic‐correctional psychiatric settings: A systematic review
Source: Psychiatry Clin Neurosci. 2026 Apr 29;80(7):546–65. doi: 10.1111/pcn.70068 (PMC13332565; doi:10.1111/pcn.70068)
Supplement: Supplementary file 1 — Table S1a. Search strategy. Table S1b. Record of Searches. Table S2. Quality assessment with NIH quality assessment tool. Table S3. Quality assessment with CASP. [file PCN-80-546-s001.docx]

Table S1a. Search strategy:

| ***Set Number*** | ***Concept*** | ***Search Statement*** |
| --- | --- | --- |
| 1 | Virtual Reality | exp Virtual Reality/ OR (virtual realit*).tw,id,kf,kw. |
| 2 | Corrections | Correctional Facilities/ OR exp Crime/ OR Criminals/ OR Prisoners/ OR (((correctional OR corrections OR penal) ADJ2 (facilit* OR institution* OR system?)) OR crime? OR criminal* OR imprison* OR incarcerat* OR inmate? OR jail* OR offender? OR prison*).tw,id,kf,kw.  NEAR/2 replaces ADJ2 in WoS |
| 3 | Forensic | Forensic Nursing/ OR Forensic Psychology/ OR exp Forensic Psychiatry/ OR (forensic? ADJ2 (assess* OR evaluat* OR facilit* OR hospital* OR inpatient? OR in-patient? OR institution* OR nurs* OR patient? OR psychiatr* OR psycholog* OR setting? OR unit?)).tw,id,kf,kw. OR forensic?.ti. |
| 4 | 2 OR 3 | |
| 5 | 1 AND 4 | |

Table S1b Record of Searches

| ***Database*** | ***Date Searched*** | ***Results*** | ***Link*** |
| --- | --- | --- | --- |
| *Medline Ovid* | Apr 21, 2024; Dec 21, 2025* | 164; 213* | <https://libaccess.mcmaster.ca/login?url=http://ovidsp.ovid.com/ovidweb.cgi?T=JS&NEWS=N&PAGE=main&SHAREDSEARCHID=37QQw9LgEM8wJcrWxsuUc10SLk2snxeb5k9vOQWNX9ClsKITTZsMad3e9aETRauU9> |
| *PsychInfo Ovid* | Apr 26, 2024; Dec 21, 2025* | 254; 321* | <https://libaccess.mcmaster.ca/login?url=http://ovidsp.ovid.com/ovidweb.cgi?T=JS&NEWS=N&PAGE=main&SHAREDSEARCHID=3Cz4dukAOf1P7haLqclIz0lh7tfyP4HnRHb9Cl2BD6H1YOHBRGdYV6FgvqR3N924D> |
| *Embase* | Apr 30, 2024; Dec 21, 2025* | 274; 346* | <https://libaccess.mcmaster.ca/login?url=http://ovidsp.ovid.com/ovidweb.cgi?T=JS&NEWS=N&PAGE=main&SHAREDSEARCHID=6sP0Ixk5fG4zZkc0EP3MuL2dHSfoquFQcO0otnrpwegxS5ikE8abWg1s8UkFNHkr3> |
| *Web of Science* | May 13, 2024; Dec 21, 2025* | 305; 437* | <https://www.webofscience.com/wos/woscc/summary/8e1aa0cd-a0b9-4c1d-9148-ceb80babe86b-e87bec15/relevance/1> |

**Legend:** The * symbol denotes the final search

Table S2. Quality assessment with NIH quality assessment tool

| Study | Study Quality Assessment Tools criteria | | | | | | | | | | | | | | | | | |
| --- | --- | --- | --- | --- | --- | --- | --- | --- | --- | --- | --- | --- | --- | --- | --- | --- | --- | --- |
|  | SD | R | 1 | 2 | 3 | 4 | 5 | 6 | 7 | 8 | 9 | 10 | 11 | 12 | 13 | 14 | score | OR |
| Fromberger et al.^40^ | CC* | 1 | Y | Y | N | N | Y | Y | NA | N | NA | Y | NR | NR | - | - | 42% | moderate |
|  |  | 2 | Y | Y | N | N | Y | Y | NA | N | NA | Y | NR | NR | - | - | 42% |  |
| Renaud et al.^38^ | CC* | 1 | Y | Y | Y | Y | Y | Y | CD | NA | NA | NA | N | NR | - | - | 50% | moderate |
|  |  | 2 | Y | Y | Y | Y | Y | Y | NA | N | NA | Y | NR | NR | - | - | 58% |  |
| Hendriks et al.^50^ | CO* | 1 | Y | Y | Y | Y | N | Y | Y | N | Y | Y | N | N | N | NR | 57% | moderate |
|  |  | 2 | Y | Y | Y | Y | N | Y | Y | N | Y | Y | N | N | NR | N | 57% |  |
| Hubal et al.^34^ | CO | 1 | Y | Y | Y | Y | N | Y | Y | N | Y | Y | Y | N | Y | N | 71% | moderate |
|  |  | 2 | Y | Y | Y | Y | N | Y | Y | N | Y | Y | Y | N | N | N | 64% |  |
| McLauchlan & Farley^42^ | CO* | 1 | Y | Y | NR | Y | N | Y | Y | N | Y | N | Y | N | N | N | 57% | moderate |
|  |  | 2 | Y | N | NR | Y | N | Y | Y | N | N | N | Y | N | N | N | 36% |  |
| Moreno et al.^53^ | CO | 1 | Y | Y | Y | Y | N | Y | Y | NA | NA | N | Y | N | Y | CD | 58% | moderate |
|  |  | 2 | Y | Y | Y | Y | N | Y | Y | NA | Y | N | Y | N | Y | N | 64% |  |
| Renaud et al.^36^ | CO* | 1 | Y | Y | CD | Y | N | Y | Y | NA | Y | N | Y | N | NA | NR | 50% | moderate |
|  |  | 2 | Y | Y | NR | Y | N | Y | Y | N | Y | N | Y | N | NA | N | 50% |  |
| Wit-De Visser et al.^58^ | CO | 1 | Y | Y | NR | Y | Y | Y | Y | NA | Y | N | Y | N | Y | N | 64% | moderate |
|  |  | 2 | Y | Y | NR | Y | Y | Y | NA | NA | Y | N | Y | N | NA | N | 50% |  |
| Seinfeld et al.^41^ | NRCT/CIS | 1 | N | N | N | N | NR | Y | Y | Y | Y | NR | Y | NR | NR | NA | 36% | low |
|  |  | 2 | N | NA | N | N | NR | Y | Y | Y | Y | NR | Y | NR | Y | NA | 43% |  |
| Seinfeld et al.^54^ | NRCT/CIS | 1 | N | NA | N | N | NR | Y | Y | NA | NR | Y | Y | NR | N | N | 29% | low |
|  |  | 2 | N | NA | N | N | NR | Y | Y | N | NR | Y | Y | NR | Y | NA | 36% |  |
| Sappelli et al.^59^ | PP† | 1 | Y | Y | NR | Y | Y | Y | Y | NA | Y | N | Y | N | - | - | 67% | moderate |
|  |  | 2 | Y | Y | Y | CD | N | Y | Y | N | Y | Y | Y | NA | - | - | 67% |  |
| van Gelder et al.^47^ | PP | 1 | N | NA | N | N | N | NA | Y | NA | Y | Y | Y | N | Y | N | 36% | low |
|  |  | 2 | N | NA | N | N | N | NA | Y | NA | Y | Y | Y | N | Y | NA | 36% |  |
| Wijk et al.^35^ | CO* | 1 | Y | Y | Y | Y | N | Y | Y | N | Y | Y | Y | N | Y | N | 71% | moderate |
|  |  | 2 | Y | Y | Y | Y | N | Y | Y | N | Y | N | Y | N | Y | N | 64% |  |
| Woicik et al.^56^ | MM*  (PP) | 1 | Y | Y | Y | Y | N | Y | Y | N | Y | Y | Y | N | N | N | 64% | moderate |
|  |  | 2 | Y | Y | Y | Y | N | Y | Y | N | Y | Y | Y | N | N | N | 64% |  |
| Smeijers et al.^45^ | RCT* | 1 | Y | Y | Y | CD | CD | CD | Y | Y | Y | Y | Y | N | Y | Y | 71% | moderate |
|  |  | 2 | Y | Y | Y | Y | Y | Y | N | Y | Y | Y | Y | N | Y | N | 79% |  |
| Smith et al.^55^ | RCT* | 1 | Y | Y | N | N | Y | Y | Y | Y | CD | Y | Y | N | Y | Y | 71% | moderate |
|  |  | 2 | Y | Y | Y | N | Y | Y | N | N | Y | Y | Y | N | Y | Y | 71% |  |
| Smith et al.^60^ | RCT | 1 | Y | Y | N | N | Y | Y | Y | N | Y | Y | Y | N | N | Y | 64% | moderate |
|  |  | 2 | Y | Y | CD | N | Y | Y | Y | Y | Y | Y | Y | N | Y | Y | 79% |  |
| Tuente et al.^43^ | RCT | 1 | Y | Y | Y | N | N | Y | N | Y | Y | Y | Y | Y | Y | Y | 79% | high |
|  |  | 2 | Y | Y | Y | N | N | Y | N | Y | Y | Y | Y | Y | Y | Y | 79% |  |
| Alshaer^48^ | PP | 1 | Y | Y | Y | CD | CD | Y | Y | N | CD | Y | N | NA | - | - | 50% | moderate |
|  |  | 2 | Y | Y | Y | CD | CD | Y | Y | N | CD | Y | N | N | - | - | 50% |  |
| Collins et al.^44^ | CS* | 1 | Y | N | NR | Y | Y | Y | N | N | Y | - | - | - | - | - | 55% | moderate |
|  |  | 2 | Y | N | NR | Y | Y | Y | N | N | Y | - | - | - | - | - | 55% |  |
| Ivarsson et al.^51^ | PP* | 1 | Y | Y | Y | N | N | Y | Y | NA | Y | Y | Y | Y | - | - | 75% | high |
|  | PP* | 2 | Y | Y | Y | N | N | Y | Y | Y | Y | Y | Y | Y | - | - | 83% |  |
| Bacon et al.^37^ | MM (CSS) | 1 | Y | Y | NR | Y | Y | Y | N | N | Y | - | - | - | - | - | 67% | moderate |
|  |  | 2 | Y | Y | NR | Y | Y | Y | N | N | Y | - | - | - | - | - | 67% |  |
| Claborn et al.^57^ | MM (PP) | 1 | Y | Y | Y | Y | N | Y | Y | NA | Y | Y | N | NA | - | - | 66% | moderate |
|  |  | 2 | Y | Y | Y | Y | N | Y | Y | N | Y | Y | N | N | - | - | 66% |  |
| Haneveld et al.^61^ | MM  (PP) | 1 | Y | Y | CD | Y | N | Y | Y | NA | Y | Y | N | NA | - | - | 58% | moderate |
|  |  | 2 | Y | Y | N | CD | N | Y | Y | N | Y | Y | Y | NA | - | - | 58% |  |
| Hedstrom et al.^49^ | MM* (PP) | 1 | Y | Y | Y | CD | N | Y | Y | NA | Y | Y | N | N | - | - | 58% | moderate |
|  |  | 2 | Y | Y | Y | Y | N | N | Y | Y | Y | Y | Y | N | - | - | 75% | moderate |
| van Rijn et al.^39^ | MM* (PP) | 1 | Y | Y | Y | N | N | Y | Y | NA | N | Y | N | NA | - | - | 50% | moderate |
|  |  | 2 | Y | Y | Y | N | N | Y | Y | CD | CD | Y | N | NA | - | - | 50% |  |

**Legend:** The * symbol denotes a pilot/feasibility/preliminary/exploratory study. 1–14 are quality assessment items/criteria, CC=Case-Control, CD=cannot determine, CIS = controlled intervention studies, CO=Cohort-Observational, CS=Case Study, CSS=Case Series Study, MM=Mixed-Methods, N=No, NA=not applicable, NIH=National Institute of Health, NR=not reported, NRCT=Non-Randomized Control Trial, OR=overall rating, PP=prepost studies with no control group, QS=Qualitative Study, R=rater, RCT=Randomized Control Trial, SD=study design, Y=Yes

Table S3. Quality assessment with CASP

| Study | Study Quality Assessment Tools criteria | | | | | | | | | | | | | |
| --- | --- | --- | --- | --- | --- | --- | --- | --- | --- | --- | --- | --- | --- | --- |
|  | SD | R | 1 | 2 | 3 | 4 | 5 | 6 | 7 | 8 | 9 | 10 | score | OR |
| Woicik et al.^56^ | MM* | 1 | Y | Y | Y | Y | Y | N | Y | N | Y | Y | 80% | high |
|  |  | 2 | Y | Y | Y | Y | Y | N | Y | N | Y | Y | 80% |  |
| Bacon et al.^37^ | MM | 1 | Y | Y | Y | N | Y | Y | N | Y | Y | Y | 80% | high |
|  |  | 2 | Y | Y | Y | Y | Y | N | Y | N | Y | Y | 80% |  |
| Claborn et al.^57^ | MM | 1 | Y | Y | Y | Y | Y | N | Y | Y | Y | Y | 90% | high |
|  |  | 2 | Y | Y | Y | Y | Y | N | Y | Y | Y | Y | 90% |  |
| Haneveld et al.^61^ | MM | 1 | Y | Y | Y | Y | Y | N | Y | Y | Y | Y | 90% | high |
|  |  | 2 | Y | Y | Y | Y | Y | N | Y | Y | Y | Y | 90% |  |
| Hedstrom et al.^49^ | MM* | 1 | Y | Y | Y | Y | Y | N | Y | Y | Y | Y | 90% | high |
|  |  | 2 | Y | Y | Y | Y | Y | Y | Y | Y | Y | Y | 100% |  |
| van Rijn et al.^39^ | MM* | 1 | Y | Y | Y | Y | Y | N | Y | N | Y | Y | 80% | moderate |
|  |  | 2 | Y | Y | Y | N | Y | N | Y | N | Y | Y | 70% |  |
| González Moraga et al.^62^ | QS | 1 | Y | Y | Y | Y | Y | N | Y | N | Y | Y | 80% | high |
|  |  | 2 | Y | Y | Y | Y | Y | N | Y | Y | Y | Y | 90% |  |
| Haneveld et al.^52^ | QS | 1 | Y | Y | Y | Y | Y | Y | Y | Y | Y | Y | 100% | high |
|  |  | 2 | Y | Y | Y | Y | Y | Y | Y | Y | Y | Y | 100% |  |
| Ivarsson et al.^63^ | QS† | 1 | Y | Y | Y | N | Y | N | Y | Y | Y | Y | 80% | high |
|  |  | 2 | Y | Y | Y | Y | Y | CD | Y | Y | Y | Y | 90% |  |
| Kouijzer et al.^64^ | QS | 1 | Y | Y | Y | N | Y | N | Y | Y | Y | Y | 80% | high |
|  |  | 2 | Y | Y | Y | N | Y | N | Y | Y | Y | Y | 80% |  |
| Mason et al.^46^ | QS | 1 | Y | Y | Y | Y | Y | CD | N | Y | Y | Y | 80% | moderate |
|  |  | 2 | Y | Y | Y | N | Y | N | N | Y | Y | Y | 70% |  |
| Sivermo et al.^65^ | QS† | 1 | Y | Y | Y | N | Y | N | Y | Y | Y | Y | 80% | high |
|  |  | 2 | Y | Y | Y | Y | Y | N | Y | Y | Y | Y | 90% |  |

**Legend:** The * symbol denotes a pilot/feasibility/preliminary/exploratory study. 1–10 are quality assessment items/criteria, CD=cannot determine, MM=mixed methods studies, N=No, NA=not applicable, NR=not reported, OR=overall rating, QS=qualitative study, R=rater; SD=study design, Y=Yes
